# Critical illness at the emergency department of a Tanzanian national hospital in a three-year period 2019–2021

**DOI:** 10.1186/s12873-023-00858-y

**Published:** 2023-08-08

**Authors:** Erick A. Mboya, Harrieth P. Ndumwa, Davis E. Amani, Paulina N. Nkondora, Victoria Mlele, Happines Biyengo, Ramadhan Mashoka, Rashan Haniffa, Abi Beane, Juma Mfinanga, Bruno F. Sunguya, Hendry R. Sawe, Tim Baker

**Affiliations:** 1 School of Public Health and Social Sciences, Dar es Salaam, Muhimbili University of Health and Allied Sciences, Dar es Salaam, Tanzania; 2 Emergency Medicine Department, Dar es Salaam, Muhimbili National Hospital, Dar es Salaam, Tanzania; 3 Mahidol Oxford Tropical Medicine Research Unit, Bangkok, Thailand; 4 Centre for Inflammation Research, University of Edinburgh, Edinburgh, UK; 5 University College London Hospitals, London, UK; 6 Department of Emergency Medicine, Muhimbili University of Health and Allied Sciences, Dar es Salaam, Tanzania; 7 Department of Clinical Research, London School of Hygiene and Tropical Medicine, London, UK; 8 Department of Global Public Health, Karolinska Institutet, Stockholm, Sweden

**Keywords:** Critical illness, Emergency department, Tanzania

## Abstract

**Background:**

Critically ill patients have life-threatening conditions requiring immediate vital organ function intervention. But, critical illness in the emergency department (ED) has not been comprehensively described in resource-limited settings. Understanding the characteristics and dynamics of critical illness can help hospitals prepare for and ensure the continuum of care for critically ill patients. This study aimed to describe the pattern and outcomes of critically ill patients at the ED of the National Hospital in Tanzania from 2019 to 2021.

**Methodology:**

This hospital-records-based retrospective cohort study analyzed records of all patients who attended the ED of Muhimbili National Hospital between January 2019 and December 2021. Data extracted from the ED electronic database included clinical and demographic information, diagnoses, and outcome status at the ED. Critical illness in this study was defined as either a severe derangement of one or more vital signs measured at triage or the provision of critical care intervention. Data were analyzed using Stata 17 to examine critical illnesses’ burden, characteristics, first-listed diagnosis, and outcomes at the ED.

**Results:**

Among the 158,445 patients who visited the ED in the study period, 16,893 (10.7%) were critically ill. The burden of critical illness was 6,346 (10.3%) in 2019, 5,148 (10.9%) in 2020, and 5,400 (11.0%) in 2021. Respiratory (18.8%), cardiovascular (12.6%), infectious diseases (10.2%), and trauma (10.2%) were the leading causes of critical illness. Most (81.6%) of the critically ill patients presenting at the ED were admitted or transferred, of which 11% were admitted to the ICUs and 89% to general wards. Of the critically ill, 4.8% died at the ED.

**Conclusion:**

More than one in ten patients attending the Tanzanian National Hospital emergency department was critically ill. The number of critically ill patients did not increase during the pandemic. The majority were admitted to general hospital wards, and about one in twenty died at the ED. This study highlights the burden of critical illness faced by hospitals and the need to ensure the availability and quality of emergency and critical care throughout hospitals.

**Supplementary Information:**

The online version contains supplementary material available at 10.1186/s12873-023-00858-y.

## Introduction

Critically ill patients have potentially reversible life-threatening conditions requiring immediate vital organ function treatment [1, 2]. Despite the potential of underestimation due to the assumption that critically ill patients are found only in the ICU, critical illness is thought to affect up to 45 million people worldwide in 2010 [3, 4]. This burden is unevenly distributed worldwide, with lower-income countries bearing the biggest brunt [3, 5]. The burden is expected to increase due to the epidemiological transition of diseases coupled with emerging and re-emerging infectious diseases [6, 7].

Critically ill patients usually present to hospitals through emergency departments (ED). At the EDs, patients are triaged according to their severity, resuscitated, and stabilized to prevent death or other complications; and transferred to the respective specialty of care [8]. With the growing field of Emergency medicine globally, low-and-middle-income countries are seeing the development of EDs in different levels of health facilities [1, 7, 9, 10].

Tanzania is no exception. Tanzania’s is a lower-middle income country and its first full-capacity public ED was established in 2010 at Muhimbili National Hospital [11]. The country is continuing to scale-up EDs that are managing an increasing number of patients presenting with diverse conditions, both critically and non-critically ill. However, the burden and characteristics of patients with critical illnesses in Tanzanian EDs have not been systematically documented. The available evidence is from studies conducted over a short period and excluded specific populations, such as children and trauma patients, who also constitute a significant proportion of ED visits [12]. Furthermore, the emergence of the COVID-19 pandemic has resulted in critical illness in about a third of its hospitalized victims, and it has radically changed health-seeking behaviors worldwide [13–15]. Recognizing the vital role of EDs as entry points to the hospitals for all patients, including children and trauma patients, and the need for a comprehensive and updated description of critical illness at the ED in resource-limited settings, especially in the era of COVID-19, this study aimed to describe the burden, trends, characteristics, causes, and outcomes of critically-ill patients at the ED of the National Hospital in Tanzania from 2019 to 2021.

## Methods

### Study design

This was a hospital-records-based cohort study of critically ill patients who attended the ED of a Tanzanian National Hospital for three years, from January 2019 to December 2021.

### Setting

The study was conducted at the Muhimbili National Hospital (MNH) ED in Dar es Salaam, Tanzania. MNH has two campuses; for this study, only data for the Upanga campus has been analyzed. The ED receives acute patients from all hospitals in the country and directly from the community and admits them at MNH or transfers them to the specialized national hospitals of Jakaya Kikwete Cardiac Institute (JKCI) or the Muhimbili Orthopedic Institute (MOI) after being triaged, stabilized and initiated management accordingly. Obstetric emergencies are handled in the obstetric unit. Trauma cases seen at the ED are transferred to MOI or MNH for further management, depending on the nature of their injury. The three hospitals, MNH, JKCI, and MOI, are located within the same campus and have a total bed capacity of 1,982 beds.

At the ED, patients are received, registered, and triaged based on their vital signs and illness severity and then managed accordingly. The goal is for stable patients to leave the ED within two hours and up-to seven hours for severely sick patients. In addition, resuscitation rooms and a mini-ICU at the ED receive severely sick patients that require emergency or critical care. Further description of the ED and the Tanzania’s health system have been described elsewhere [11, 16].

Dar es Salaam is the most populous city in Tanzania. During the COVID-19 pandemic, over 40,000 confirmed cases have been reported in Tanzania, with Dar es Salaam having the highest number of cases [17, 18].

### Data source

Data were extracted from the ED electronic information system. Clinicians and triage nurses at the ED enter information about the patients into the electronic system as they are providing care. The extracted data included the time the patient arrived at and was discharged from the ED, demographic information, the presenting complaint, diagnoses at the ED (the first listed diagnosis was used as the main diagnosis), interventions provided, laboratory results, ED outcome status, and the corresponding admission ward at MNH or transfer facility.

### Study population and eligibility criteria

All patients who presented to the ED within three years, from 1st January 2019 to 31st December 2021, were included in the study. Critical illness was primarily defined as present in any patient who *either* had a severe derangement of one or more vital signs as measured at triage *or* received a critical care intervention at the ED. The critical interventions included oxygen therapy, intubation, suction, tracheostomy, cardiopulmonary resuscitation, adrenaline/epinephrine, atropine, and chest tube insertion. The vital signs included systolic blood pressure (SBP), pulse rate (HR), respiratory rate (RR), blood oxygen saturation (SpO2%), and the Glasgow coma scale (GCS). The age-dependent cut-offs for severe derangement of vital signs were based on previous literature (Table 1) [1, 19–22].

**Table 1:** Cut-offs for severely deranged vital signs by age

Vital Sign	Age	Severely Deranged Cut-off
Respiratory rate per minute	< 1 month	< 20 or > 80
	1 month-<1 year	< 15 or > 60
	1 year- <5 years	< 10 or > 50
	5 years- 12 years	< 8 or > 40
	> 12 years	< 8 or > 30
Saturation (%)	All	< 90%
Pulse rate per minute	< 1 month	< 80 or > 200
	1 month- <1 year	< 80 or > 180
	1 year- <5 years	< 70 or > 170
	5 years- 12 years	< 60 or > 150
	> 12 years	< 40 or > 130
Glasgow Coma Scale	All	≤ 8/15
Systolic Blood Pressure (mmHg)	< 3 months	< 50
	3 months- <1 year	< 70
	1 year- <4 years	< 75
	4 years- <12 years	< 80
	≥ 12 years	< 90

Because critical illness is notoriously difficult to define [2, 4], and due to the limitations of our data source, retrospective data collected primarily for clinical purposes, we used two additional, alternative definitions to estimate the burden of critical illness in sensitivity analyses. First, we used a broader definition for critical illness, including patients who received care in the resuscitation room or the mini-ICU in the ED in addition to those in the primary definition. The second alternative definition was more restrictive and included only patients with one or more severely deranged vital signs.

**Fig. 1:**
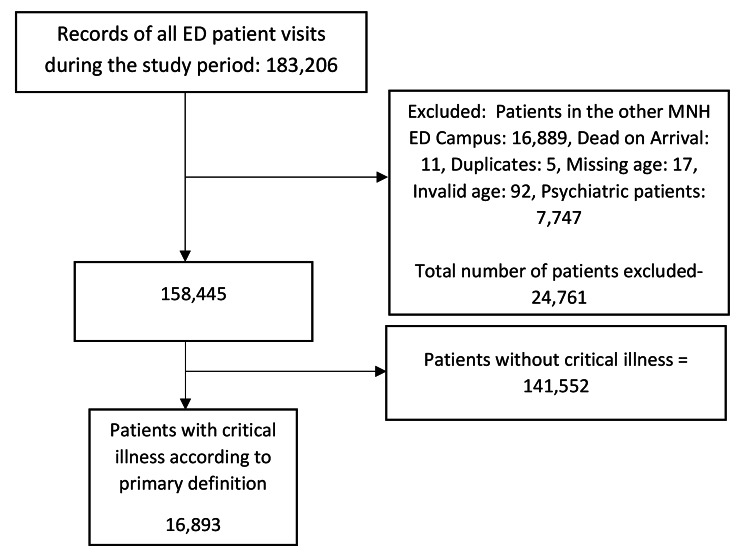
Flowchart of the data extraction for the study participants

We excluded from this study records of patients who lacked data for their age or were dead at arrival. Psychiatric patients with non-somatic complaints were also excluded, as were those in the electronic information system but had received care in the ED of MNH’s additional campus (Fig. 1).

### Data extraction, management, and analysis

Anonymized data were extracted from the electronic information system in Excel format and were imported into STATA 17 (StataCorp, Texas, USA). Eligible patients were identified, and duplicates were removed. The data were cleaned and checked for their validity and completeness, and outliers were identified and cleaned or removed if not plausible. Descriptive statistics using frequencies, percentages, median and interquartile range (IQR) were used to summarize the patients’ demographic and clinical characteristics.

Missing vital signs were imputed as not being severely deranged. A sensitivity analysis where missing vital signs were imputed as severely deranged was done. Imputation was not done for the Glasgow Coma Scale for all patients and not for systolic blood pressure for children under 15 years, as there was significant non-random missing data.

The burden of critical illness, defined by the primary and alternative definitions and the sensitivity analysis, was calculated and presented. Using the primary definition, the total number and proportion of critically ill patients per year and their mean daily/monthly numbers were calculated. The trend of the monthly proportion of patients who were critically ill was presented with line graphs, together with the number of critically ill, non-critically ill and total number of patients. The proportion of critically ill patients were further disaggregated by the demographic of the patients. The proportion of critical illness by year was done, and the chi-squared test was used to compare the observed proportion of critical illness in 2020 and 2021 to that in the first year, 2019. Diagnoses of critically ill patients were classified according to the first listed diagnosis and grouped using the organ system affected. Outcomes at the ED were presented using pie charts, and admission wards were categorized and presented using bar graphs.

## Results

There were 158,445 patient visits to the ED between January 2019 and December 2021. On average, 4,401 patient visits were made per month – 144.6 per day. The median age (IQR) of patients who attended the ED was 34.6 (21.0- 54.1) years. About 60% of all patients were below 45 years of age, and over half (55%) were men. Most of the patients (80.9%) who attended the ED came from Dar es Salaam and the neighboring regions in the Eastern zone. Less than half of the patients (46.8%) had health insurance coverage, and 14.9% of the patients were brought in using an ambulance (Table 2).

**Table 2:** Demographic characteristics of patients who attended the MNH ED 2019–2021

Variable	2019 (61,815)	2020 (47,361)	2021 (49,269)	Overall (158,445)
	n (%)	n (%)	n (%)	N (%)
**Age group**				
0- <15 years	11,993 (19.4)	9452 (20.0)	9645 (19.6)	31,090 (19.6)
15- <30 years	14,628 (23.7)	10,295 (21.7)	10,319 (20.9)	35,242 (22.2)
30- <45 years	13,441 (21.7)	10,544 (22.3)	11,077 (22.5)	35,062 (22.1)
45- <60 years	10,761 (17.4)	8161 (17.2)	8734 (17.7)	27,656 (17.5)
60- <75 years	8008 (13.0)	6487 (13.7)	6919 (14.0)	21,414 (13.5)
75-<90 years	2735 (4.4)	2189 (4.6)	2356 (4.8)	7280 (4.6)
>=90 years	249 (0.4)	233 (0.5)	219 (0.4)	701 (0.4)
**Sex**				
Male	33,801 (54.7)	25,968 (54.8)	27,331 (55.5)	87,100 (55.0)
Female	28,014 (45.3)	21,393 (45.2)	21,938 (44.5)	71,345 (45.0)
**Zones**				
Dar Es Salaam & Eastern Zone	49,852 (80.6)	38,033 (80.3)	40,279 (81.8)	128,164 (80.9)
Elsewhere	11,963 (19.4)	9328 (19.7)	8990 (18.2)	30,281 (19.1)
**Mode of Arrival**				
Ambulance	8662 (14.0)	7300 (15.4)	7665 (15.6)	23,627 (14.9)
Public transport	24,893 (40.3)	18,115 (38.3)	17,412 (35.3)	60,420 (38.1)
Private car/ Taxi	18,745 (30.3)	15,856 (33.5)	16,716 (33.9)	51,317 (32.4)
Walked	3592 (5.8)	2496 (5.3)	2258 (4.6)	8346 (5.3)
Others*	498 (0.8)	594 (1.3)	311 (0.6)	1403 (0.9)
Missing	5425 (8.8)	3000 (6.3)	4906 (10.0)	13,331 (8.4)
**Payment Mode**				
Public/ Cost sharing	26,896 (43.5)	22,122 (46.7)	22,948 (46.6)	71,966 (45.4)
Health Insurance	30,456 (49.3)	21,194 (44.7)	22,527 (45.7)	74,177 (46.8)
Private Payment	2614 (4.2)	2375 (5.0)	2269 (4.6)	7258 (4.6)
Others**	162 (0.3)	102 (0.2)	249 (0.5)	513 (0.3)
Missing	1687 (2.7)	1568 (3.3)	1276 (2.6)	4531 (2.9)

* *stretchers, carried by good samaritans*

** *Exemption, foreigners*

**Table 3:** Clinical characteristics of patients who attended the MNH ED 2019–2021

Variable	2019 (61,815)	2020 (47,361)	2021 (49,269)	Overall (158,445)*
	n (%)	n (%)	n (%)	N (%)
**Severely Deranged SBP**				
No	47,023 (76.1)	36,617 (77.3)	38,574 (78.3)	122,214 (77.1)
Yes	1052 (1.7)	716 (1.5)	760 (1.5)	2528 (1.6)
Missing	13,740 (22.2)	10,028 (21.2)	9935 (20.2)	33,703 (21.3)
**Severely Deranged RR**				
No	54,390 (88.0)	42,407 (89.5)	42,504 (86.3)	139,301 (87.9)
Yes	981 (1.6)	977 (2.1)	825 (1.7)	2783 (1.8)
Missing	6444 (10.4)	3977 (8.4)	5940 (12.1)	16,361 (10.3)
**Severely Deranged HR**				
No	53,636 (86.8)	42,049 (88.8)	42,148 (85.5)	137,833 (87.0)
Yes	2498 (4.0)	1963 (4.1)	1869 (3.8)	6330 (4.0)
Missing	5681 (9.2)	3349 (7.1)	5252 (10.7)	14,282 (9.0)
**Severely Deranged SpO2**				
No	54,832 (88.7)	42,870 (90.5)	42,757 (86.8)	140,459 (88.6)
Yes	1396 (2.3)	1245 (2.6)	1384 (2.8)	4025 (2.5)
Missing	5587 (9.0)	3246 (6.9)	5128 (10.4)	13,961 (8.8)
**Severely Deranged GCS**				
No	8259 (13.4)	7754 (16.4)	7759 (15.7)	23,772 (15.0)
Yes	311 (0.5)	232 (0.5)	238 (0.5)	781 (0.5)
Missing	53,245 (86.1)	39,375 (83.1)	41,272 (83.8)	133,892 (84.5)
**Received Critical Care Intervention**				
No	55,786 (90.2)	42,673 (90.1)	42,109 (85.5)	140,568 (88.7)
Yes	1966 (3.2)	1648 (3.5)	1861 (3.8)	5475 (3.5)
Missing	4063 (6.6)	3040 (6.4)	5299 (10.8)	12,402 (7.8)

*SBP- Systolic Blood Pressure, RR- Respiratory rate, HR- Heart rate, GCS- Glasgow Coma Scale, SpO2- Oxygen Saturation*

* The sum of severely deranged individual vital signs is more than the total number of critically ill patients as patients could have more than one severely deranged vital sign

### The burden of critical illness

**Fig. 2:**
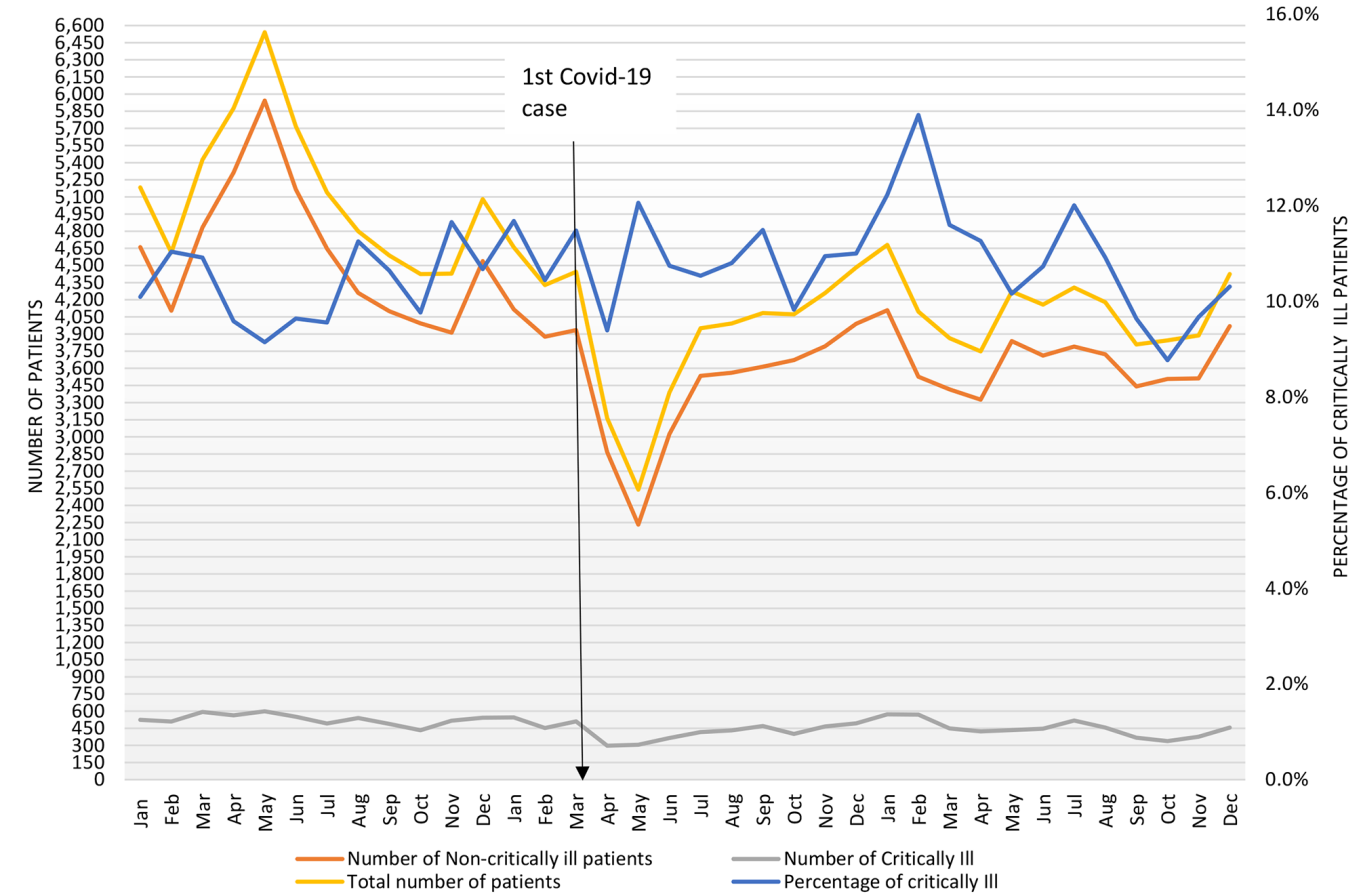
Monthly trend of critical illness in the MNH ED from January 2019 to December 2021. Includes the proportion of patients who were critically ill and the absolute numbers of critically ill, non-critically ill, and total patients who attended the ED each month since January 2019

According to the primary definition, 16,893 (10.7%) patients were critically ill. These include 5475 patients (3.5%) classified as critically ill as they received critical care interventions and 13,893 patients (8.8%) classified as critically ill as they had one or more severely deranged vital signs (Table 3). The number and proportion of patients at the ED who were critically ill were 6,345 (10.3%) for 2019, 5,148 (10.9%) for 2020 (*X*^*2*^ p-value = 0.001), and 5,400 (11.0%) for 2021 (*X*^*2*^ p-value < 0.001) (Fig. 2). The mean number of critically ill patients seen per month at the ED was 481 (ranging from 297 to 598, mean per day = 15.4).

Using the alternative definitions, the burden of critically-ill patients was 8.8% for those with severely deranged vitals alone and 30.8% when patients who received care in the resuscitation rooms or the mini ICUs were also included. When patients with missing vitals were imputed as critically ill, the estimated burden was 21.0%.

### The burden of critical illness by socio-demographic characteristics

The median age (IQR) of critically ill patients was 36 (15.1–57.9) years. Males comprised slightly over half (54.4%) of the critically ill patients, and about a quarter (24.8%) were children under the age of 15 years. About a third (34.3%) of critically ill patients had health insurance, and over a third (37.1%) of them were brought to the ED by ambulance.

The proportion critically ill patients among male and female patients attending the MNH ED was 10.6% and 10.8%, respectively. The proportion of critically ill patients was highest among patients attending the ED who were older than 90 years (17.4%), those 75–90 years (13.8%), and children under 15 years (13.5%). About one in ten patients from other regions (11.9%) were critically ill, similarly for patients coming from Dar es Salaam and the Eastern zone (10.4%). The proportion of critical illness was highest among patients who were paying out-of-pocket (10.9%) and on public/cost-sharing plans (13.7%) and was lowest among patients with health insurance (7.8%). About one in four patients, (26.6%), brought by an ambulance were critically ill —Table 4.

**Table 4:** Distribution and prevalence of critically ill patients by demographic characteristics

Variable	2019 (N = 6345)	2020 (N = 5148)	2021 (N = 5400)	Overall (N = 16,893)	Proportion of critically ill (%)
	n (%)	n (%)	n (%)	n (%)	
**Median age in years (IQR)**	34.7 (14.0, 56.0)	36.6 (15.4, 58.5)	39 (16.5, 59.2)	36.47 (15.1, 58)	
**Age groups**					
0–15 years	1628 (25.7)	1264 (24.6)	1295 (24.0)	4187 (24.8)	13.5
15- <30 years	1146 (18.1)	865 (16.8)	832 (15.4)	2843 (16.8)	8.1
30- <45 years	1196 (18.8)	938 (18.2)	964 (17.9)	3098 (18.3)	8.8
45- <60 years	1037 (16.3)	878 (17.1)	997 (18.5)	2912 (17.2)	10.5
60- <75 years	926 (14.6)	858 (16.7)	939 (17.4)	2723 (16.1)	12.7
75-<90 years	364 (5.7)	308 (6.0)	336 (6.2)	1008 (6.0)	13.8
>=90 years	48 (0.8)	37 (0.7)	37 (0.7)	122 (0.7)	17.4
**Sex**					
Male	3470 (54.7)	2754 (53.5)	2971 (55.0)	9195 (54.4)	10.6
Female	2875 (45.3)	2394 (46.5)	2429 (44.9)	7698 (45.6)	10.8
**Zone**					
Dar Es Salaam & Eastern zone	4996 (78.7)	4050 (78.7)	4344 (80.4)	13,390(79.3)	10.4
Elsewhere	1349 (21.3)	1098 (21.3)	1056 (19.6)	3503 (20.7)	11.6
**Payment Mode**					
Public/ Cost sharing	2298 (36.2)	1915 (37.2)	2062 (38.2)	6275 (37.1)	13.7
Health Insurance	1579 (24.9)	1224 (23.8)	1160 (21.5)	3963 (23.5)	7.8
Private Payment	2221 (35.0)	1867 (36.3)	2058 (38.1)	6146 (36.4)	10.9
Others	154 (2.4)	74 (1.4)	76 (1.4)	304 (1.8)	9.7
Missing	63 (1.0)	67 (1.3)	38 (0.7)	168 (1.0)	9.4
**Mode of arrival**	30 (0.5)	1 (< 1)	6 (0.1)	37 (0.2)	
Ambulance					26.6
Public transport	3688 (58.1)	3093 (60.1)	3048 (56.4)	9829 (58.2)	7.1
Private car/ Tax	2226 (35.1)	1649 (32.0)	1917 (35.5)	5792 (34.3)	11.9
Walked	263 (4.1)	250 (4.9)	281 (5.2)	794 (4.7)	3.6
others	15 (0.2)	11 (0.2)	24 (0.4)	50 (0.3)	10.5
Missing	153 (2.4)	145 (2.8)	130 (2.4)	428 (2.5)	0.3

### Underlying first-listed diagnoses among critically ill patients

The five-leading diagnoses among patients with critical illness were respiratory diseases (18.8%), cardiovascular diseases (12.6%), infectious diseases and trauma contributing each 10.2%, and malignancies (8.7%) —Table 5. The list of diseases in each categories can be found in Supplement 1.

**Table 5:** Disease categories of the first-listed diagnoses of patients attended at the ED

Diseases Categories	Frequency	Percent (%)
Respiratory Diseases	3,172	18.8
Cardiovascular Diseases	2,128	12.6
Infectious diseases	1,720	10.2
Injury & Trauma	1,719	10.2
Cancers/ Malignancies	1,462	8.7
Neurological Diseases	1,291	7.6
Gastrointestinal Diseases	1,160	6.9
Renal Diseases	787	4.7
Hematological Diseases	708	4.2
Endocrine Diseases	308	1.8
Urinary tract Diseases	256	1.5
Others	2,066	12.2
Missing	116	0.7

### Outcomes of critically ill patients at the ED

**Fig. 3:**
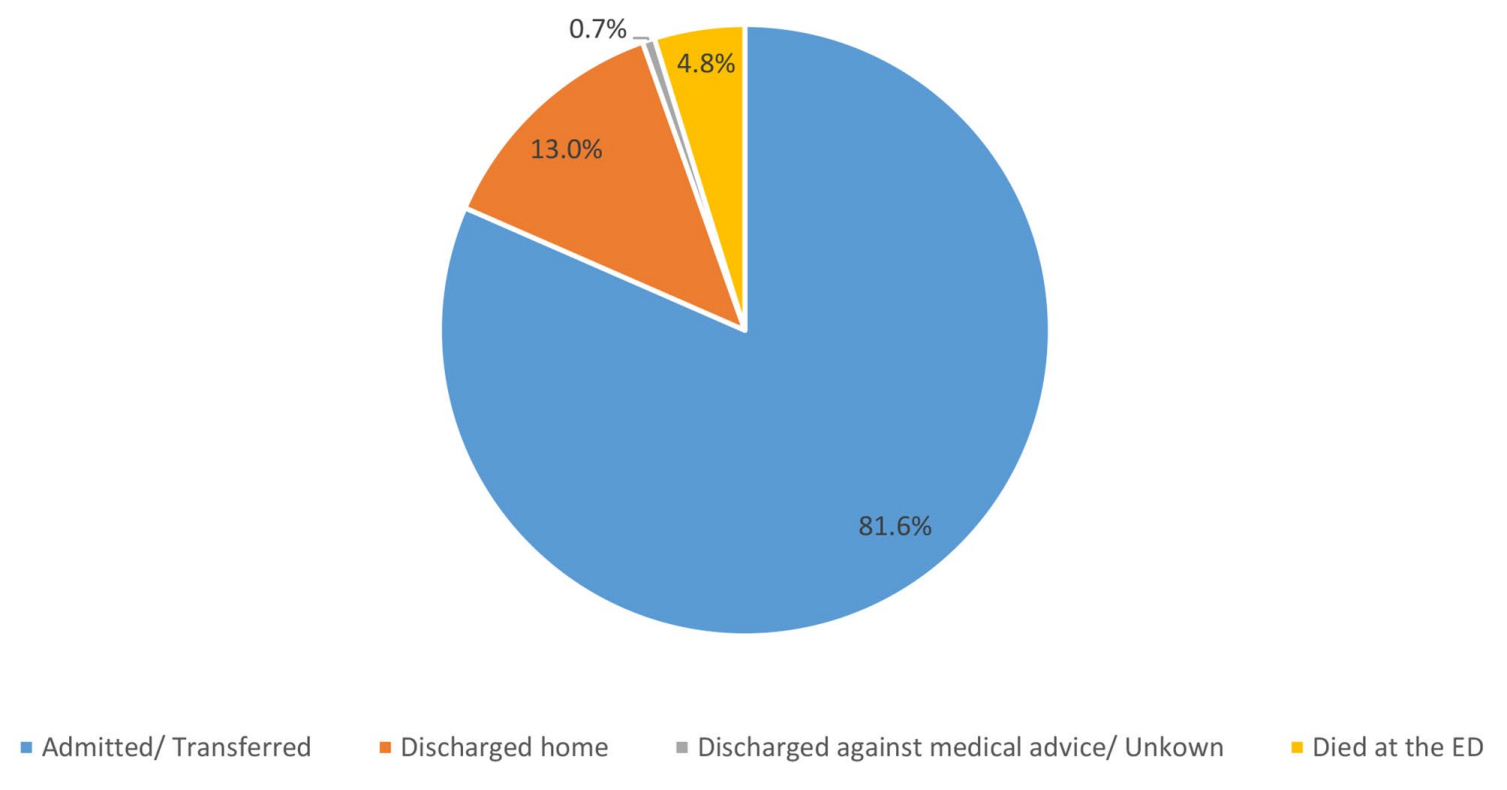
Outcome of critically ill patients at the ED (N = 16,893)

Most, 13,777 (81.6%), of the critically ill patients presenting at the ED were admitted, and 809 (4.8%) died at the ED. About 13% of the critically ill patients were discharged after treatment at the ED (Fig. 3). The proportion of critically ill patients who died in the ED was 4.2% in 2019 and 4.3% in 2020 (*X*^2^ = 0.744), and 5.9% in 2021 (*X*^2^ < 0.001).

**Fig. 4:**
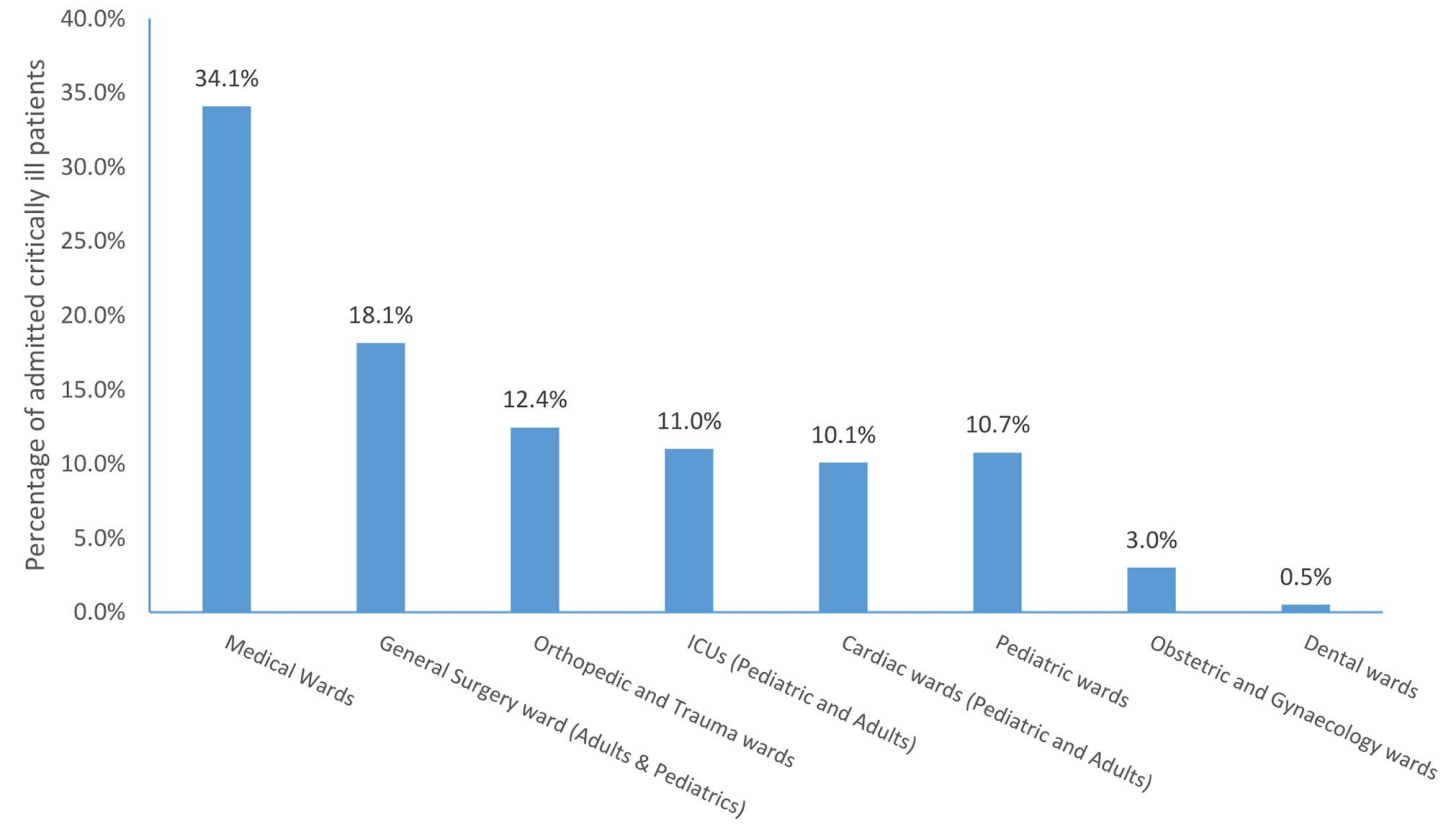
The proportion of admitted critically ill patients by specialty of care *(*N = 13,777)

Among those admitted, 11% of critically ill patients were admitted to the ICUs (Pediatric ICU, neonatal ICU, or adult ICU), and 89% were admitted to general wards (Fig. 4).

## Discussion

Critically ill patients make up about 10% of all patients attended at the ED of a national hospital in Tanzania. The COVID-19 pandemic did not lead to an increase in patient numbers – in fact the overall number of patients who attended the ED and the number of critically ill patients in 2020 and 2021 was lower than that in 2019. The major diagnoses leading to critical illness were respiratory, cardiovascular, and infectious diseases as well as trauma, and the majority of the critically ill were admitted to the hospital’s general wards.

Although the *proportion* of critically ill at the ED increased slightly in the years under review, the *number* of ED visits declined. Hence, the observed increase in the proportion of critical illness is driven by a sharper decline of non-critically ill patients than of critically ill patients. Although COVID-19 has been reported to cause critical illness in up to 30% of patients, it is striking that the start of the pandemic in Tanzania in April 2020 coincides with a decrease in patients coming to the ED. This may be due to public health responses, changes in health system policies, or changed community practices of attending the national hospital during the pandemic. Similar findings have been observed in other settings globally [13, 23, 24]. It should be noted that although Tanzania did not implement strict lockdown measures, several preventive measures were implemented in the first wave in 2020[25]. The decreasing number of critically ill patients may also be due to a decline in other conditions, such as road traffic trauma during the pandemic [26].

The proportion of critical illness observed in this study is higher than that reported in a study done previously at the same facility, describing critical illness among adults (6.9%) [12]. Their criteria for critical illness included a triage category and an early warning score cut-off which is more restrictive than in our study. Using more generous individual deranged vital signs as criteria in our study avoids missing an important proportion of patients with a higher risk of death [21].

Critically ill patients had higher ED mortality (4.8%) compared to the ED mortality of all the patients (0.6%) and much higher than non-critically ill (0.06%). The derangement of at least one physiologic parameter has been associated with higher mortality, but early recognition of the derangement and interventions has shown evidence to reduce mortality for some conditions of critically ill patients [27, 28]. The ED mortality among critically ill patients was higher in 2021 than in 2019 or 2020. The reasons for this observation are still unclear, and further investigation is warranted.

The underlying causes of critical illness were diverse. Both infectious and non-communicable diseases are prevalent in Tanzania and cause substantial burdens of critical illness [29]. Indeed, critical illness can be due to a large variety of underlying diseases and can be found in all medical specialties. Specialty or disease-based programs may find it challenging to provide quality care for critically ill patients, and thus all wards and areas of health facilities require the capacity to provide care for critically ill patients. We found that only 11% of critically ill patients were admitted to ICU, while 89% were admitted to general wards. Using ICU-treated patients as the indicator of the burden of critical illness, as has been done previously, can significantly underestimate the burden of critical illness, even in tertiary hospitals [4]. Studies have shown that there is a lack of resources for the provision of emergency and critical care in LMICs [9, 10, 30]. To increase resources and improve care, especially ward-based care for critical illness, the approach described as essential emergency and critical care (EECC) should be implemented [7, 19, 31]. EECC is defined as “the effective lifesaving care of low- cost and low- complexity that all critically ill patients should receive in all wards in all hospitals in the world”. It focuses on early identification of critical illness, timely provision of life-saving care and that is of low cost and low complexity [7, 31]. Because critically ill patients comprises a heterogeneous group of patients, EECC should be a critical part in every specialty in a health facilities, as it improves the care given to critically ill patients and substantially reduce preventable deaths [19, 28].

### Strengths and limitations of the study

This study used a single parameters rather than compound scores such as early warning scores because single parameters are easy and less time consuming when resources are limited, and avoid calculation errors. Moreover, single parameters indicate the action that could be taken (such as give oxygen if low saturation) which compound scores don’t [32]. Another strength is that this large study over three years includes a population of over 150,000 patients presenting to the hospital and almost 17,000 critically ill patients. This enables precise estimates of the burden and disaggregation of estimates. However, the study is from a single national center which is not necessarily representative of other settings in the country. Another limitation of this study is the use of hospital record data, where some vital signs data were missing and were treated as not severely deranged. As a result there is a possibility that the burden of critical illness due to deranged vital signs is underestimated. However, we also presented the estimates of the burden where missing values were imputed as severely deranged, which may overestimate the burden. Also, although the analysis of the underlying diagnoses using the clinicians’ recorded first-listed diagnosis has provided useful information, it is imperfect as it relies on the assumption that the first listed diagnosis is the most important and that diagnoses are correct – both assumptions may not hold.

## Conclusions

More than one in ten patients attending the emergency department of a Tanzanian National Hospital had a critical illness. The number of critically ill patients did not increase during the pandemic. The majority were admitted to general hospital wards, and about one in twenty died at the ED. This study highlights the burden of critical illness faced by hospitals and the need to ensure the availability and quality of emergency and critical care throughout hospitals.

### Electronic supplementary material

Below is the link to the electronic supplementary material.

Supplementary Material 1

## Data Availability

The data that support the findings of this study are available from the Muhimbili National Hospital but restrictions apply to the availability of these data, which were used under license for the current study, and so are not publicly available. However, upon request and following national policies for data sharing, data may be accessed to researchers requesting and fulfilling requirements as per the Tanzanian National Health Research Ethics guidelines. Contact information: National Health Research Ethics Review Committee Contact email: jikingura@nimr.or.tz Postal address: National Institute for Medical Research P.O. Box 9653 Dar es Salaam, Tanzania.

## Abbreviations

ED: Emergency department
EECC: Essential Emergency and Critical Care
GCS: Glasgow Coma Scale
HR: Pulse rate
ICU: Intensive Care Unit
IQR: Inter Quartile Range
JKCI: Jakaya Kikwete Cardiac Institute
MNH: Muhimbili National Hospital
MOI: Muhimbili Orthopedic Institute
MUHAS: Muhimbili University of Health and Allied Sciences
RR: Respiratory rate
SBP: Systolic Blood Pressure
SpO2: Oxygen Saturation
