# Supplementary material for: Critical illness at the emergency department of a Tanzanian national hospital in a three-year period 2019–2021
Source: BMC Emerg Med. 2023 Aug 8;23:86. doi: 10.1186/s12873-023-00858-y (PMC10408204; doi:10.1186/s12873-023-00858-y)
Supplement: Supplementary file 1 — Supplementary Material 1 [file 12873_2023_858_MOESM1_ESM.docx]

**Supplement Table 1: Major categories and the first-listed diagnoses of critically ill patients attended at the ED of a Tanzanian national hospital, 2019- 2021.**

| **Diseases Categories** | **Frequency** | **Percent (%)** |
| --- | --- | --- |
| **Respiratory Diseases** | **3,172** | **18.8** |
| *Pneumonia* | *1729* |  |
| *Tuberculosis (PTB & EPTB)* | *292* |  |
| *Pulmonary edema* | *244* |  |
| *Asthma* | *158* |  |
| *Others* | *749* |  |
| **Cardiovascular Diseases** | **2,128** | **12.6** |
| *Hypertension & Heart Diseases* | *1174* |  |
| *Congenital heart diseases* | *417* |  |
| *Shock* | *313* |  |
| *Pulmonary embolism* | *183* |  |
| *Others* | *41* |  |
| **Infectious diseases** | **1,720** | **10.2** |
| *Sepsis* | *1106* |  |
| *HIV/AIDS/STDS* | *268* |  |
| *Malaria* | *202* |  |
| *Dengue fever* | *79* |  |
| *Others* | *65* |  |
| **Injury & Trauma** | **1,719** | **10.2** |
| *TBI* | *882* |  |
| *Other trauma* | *747* |  |
| *Visceral injury* | *50* |  |
| *Snake/ Dog bite* | *40* |  |
| **Cancers/ Malignancies** | **1,462** | **8.7** |
| *Esophageal cancer* | *220* |  |
| *Abdominal tumors* | *198* |  |
| *Breast cancer* | *116* |  |
| *Lung cancer* | *113* |  |
| *Hematological malignancies* | *52* |  |
| *Other neoplasms* | *763* |  |
| **Neurological Diseases** | **1,291** | **7.6** |
| *Stroke* | *765* |  |
| *Seizure disorder* | *157* |  |
| *Meningitis* | *143* |  |
| *Cerebral palsy* | *61* |  |
| *Other neurological disorders* | *165* |  |
| **Gastrointestinal Diseases** | **1,160** | **6.9** |
| *Gastritis/ Esophagitis* | *295* |  |
| *Gastroenteritis* | *211* |  |
| *Intestinal Obstruction* | *170* |  |
| *Liver cirrhosis/failure* | *200* |  |
| *Others* | *263* |  |
| **Renal Diseases** | **787** | **4.7** |
| *Renal failure* | *654* |  |
| *Electrolyte imbalance* | *120* |  |
| *Nephrotic/Nephritic syndrome* | *13* |  |
| **Hematological Diseases** | **708** | **4.2** |
| *Anemia* | *512* |  |
| *Sickle cell disease* | *141* |  |
| *Bleeding disorder* | *55* |  |
| **Endocrine Diseases** | **308** | **1.8** |
| *Diabetes* | *236* |  |
| *Hypoglycemia* | *39* |  |
| *Thyroid Disease* | *33* |  |
| **Urinary tract Diseases** | **256** | **1.5** |
| *Prostate cancer/ enlargement* | *159* |  |
| *UTI* | *75* |  |
| *Obstructive uropathy* | *15* |  |
| *Epididymorchitis* | *7* |  |
| **Others** | **2,066** | **12.2** |
| *Malnutrition* | *191* |  |
| *Skin infections* | *148* |  |
| *Foreign body* | *131* |  |
| *Wound/Ulcer* | *131* |  |
| *Others* | *1465* |  |
| **Missing** | **116** | **0.7** |
